# Acute Yellow Atrophy of the Liver, with Report of Case

**Published:** 1886-12

**Authors:** F. R. Campbell


### Clinical Reports.

### *ACUTE YELLOW ATROPHY OF THE LIVER, WITH REPORT OF CASE.*

By F. R. CAMPBELL, A. M., M. D.

Acute yellow atrophy of the liver, or hepatitis cytophthora, is one of the rarest diseases known to man. Not more than 200 cases are recorded in all medical history. In some large hospitals the affection is not observed in years, while the great majority of physicians never meet with a case in their practice. Nevertheless cases do occur and we are liable to be called upon to treat them. Many if not most of the cases of malignant jaundice, or icterus gravis of the older writers were really cases of acute atrophy. Morgagni reported the first case, Rokitarský, in 1842, gave the first complete description of the disease, and since the publication of his observations the disease has many times been described.

In regard to the causes of acute atrophy of the liver but little is known. Statistics show that three fifths of all cases occur among females, that pregnant women are most frequently affected, and that seventy per cent. of the cases occur between the ages of twenty and forty-five. For the most part it is well-nourished persons who are attacked, and far more frequently robust than delicate individuals. Intemperance, over-feeding, and sexual excesses have all been mentioned as predisposing causes, but there are very few facts to substantiate such statements.

Of exciting causes, still less is known; depressing mental emotion, anger and fright have been enumerated in this connection. It is sometimes a secondary affection following cirrhosis or *obstruction of the bile ducts.* It sometimes complicates typhoid fever, puerperal fever, and is usually associated with phosphorus poisoning.

The pathological changes characteristic of the disease are reduction of size of the liver, its tissues are flaccid and have a saffron or rhubarb color. Sometimes the size is diminished one half or more, the reduction being due to atrophy of the hepatic cells. Extravasations of blood are found in many organs especially the stomach and intestines. The spleen is usually enlarged and often there are inflammatory changes in the kidneys and other organs.

With regard to clinical history the disease is divided into two periods, the prodromal, or mild stage, and the cholæmic, or serious stage, each being characterized by special symptoms, although in some cases the prodromal stage is apparently absent. The first symptom is usually loss of appetite followed in many cases by nausea, vomiting and constipation. Then jaundice makes its appearance, the urine is stained with bile pigments and the fæces assume a clayey color. Pain is present in the region of the liver in about one-half of all cases; fever is developed, the temperature rising and falling irregularly, but disappearing entirely at the beginning of the second stage. The pulse is at first not accelerated but increases in frequency when the fever comes on. The patient is troubled with obstinate insomnia and becomes restless, tossing and rolling in his bed. The liver is not as a rule reduced in size in this stage.

Before the second stage begins there is often an apparent improvement in the patient's condition, the temperature becomes normal and the gastric disturbances are not so marked. But the apparent improvement is only a calm before the storm. Marked nervous and psychical disturbances are suddenly developed, the pupils are dilated, there are muscular twitchings, mental excitement, hallucinations of sight and hearing, delirium, then coma, sometimes followed by convulsions and always by death. This second period has been divided by Ozonam into the stage of excitement and the stage of collapse. In the former the delirium is often of a fierce character, it is impossible to keep the patient in bed, he makes frequent attempts to escape, and often attacks his attendants. In the latter stage the pulse becomes very frequent and loses strength, the tongue becomes dry and brown, sordes collect on the teeth, the respiration is accelerated and a peculiar moaning sound is produced, the delirium becomes muttering, and with the coma stertorous breathing is observed, the quantity of urine is diminished; there may be entire suppression or retention. In the urine crystals of leucin and tyrosin are found. These may be present in the first period, but are not so abundant. Albumen is usually present; the amount of urea is reduced. Toward the close of life hemorrhages occur in various organs, especially the alimentary canal, and black vomit and fæces may be observed. The liver rapidly diminishes in size while the spleen becomes enlarged.

A positive diagnosis in the first stage is, according to all authorities, impossible, though when we have vomiting and great prostration in connection with icterus, acute atrophy of the liver may be suspected, but even these symptoms are sometimes present in simple obstructive jaundice. With the appearance of the second or toxæmic stage, the diagnosis usually becomes clear. "The icterus, the delirium and coma, the absence of fever, the diminution of hepatic dullness and the presence of leucin and tyrosin in the urine, constitute a train of symptoms repeated in no other disease" (Thierfelder). Acute phosphorus poisoning is the only affection which would render a differential diagnosis difficult.

The duration of the disease varies from four days to as many weeks, but the second stage is almost uniformly very limited.

Thirty-seven per cent. of all cases die within ten days of the appearance of the first symptoms. Of 118 cases, fifty-six died within forty-eight hours from beginning of second stage, eighty-two within three days, and ninety-nine within four days. While only one lived fourteen days. It is very questionable if the disease can terminate otherwise than fatally (Eichorst, Thierfelder, Bartholow). Four or five recoveries have been reported, but in these cases the diagnosis is doubtful. Accordingly no specific medication is possible and all remedies must be administered to meet symptomatic indications.

The writer has recently had under his care a patient whose symptoms were those of a typical case of acute yellow atrophy of liver, and although an autopsy could not be obtained to verify the diagnosis, he considers it worthy of a place in medical literature.

F. H. W., male, age 42, married, manufacturer, called at my office, Nov. 11th, to consult me regarding a severe pain in his epigastric and right hypochondriac regions. He was a dark complexioned, corpulent man of a bilious temperament. He had always been a total abstainer from alcohol, but was a large eater and fond of rich food. He had had hernia and typhoid fever twenty years ago; on recovering from the fever the hernia was found to be cured. Since the above-mentioned sickness he had enjoyed the best of health, with the exception of a slight attack of jaundice three years ago. During the past year was troubled with irritability of bladder, and occasional attacks of pain in epigastrium, which he attributed to dyspepsia, and for the relief of which he was in the habit of taking tablets of pepsin and Bethesda water. On the evening of November 8th, after eating a hearty meal, he had an attack of nausea and vomiting, but no pain. He prescribed for himself a purgative and on the morning of the 9th, said he was well, with the exception of loss of appetite. On the evening of the 10th, while riding in a sleeping car, he was attacked with a severe pain shooting from the epigastrium toward the right scapula. This pain, which he described as "most agonizing," continued during the night with only slight intermissions. He was bathed with a cold perspiration, and felt that even death was preferable to such suffering. This pain disappeared during the day, and when seen by the writer only a slight tenderness in hepatic region was observed. His eyes were jaundiced, urine gave the characteristic green color, when examined for bile by Marèchal's iodine test; Genelin's test also showed the presence of biliory acids in urine; albumen was absent. No test made for leucin and tyrosin. His pulse and temperature were normal, his tongue was furred but not dry. Suspecting that he had had an attack of biliary colic, I prescribed for him rest in bed, a nitrogenous diet, excluding all fats and carbo-hydrates, and ordered phosphate of sodium ʒ i. t. i. d., as a laxative, and acid hydrochloric dil, m. v., every four hours. In case he had another attack of pain, I directed him to send for me.

Nov. 12th, at 2 a. m., was called to patient; found him suffering from the pain described as occurring the preceding night, but not so severe. Administered morph. sulph., gr. ¼, with atropia gr. 1-150 subcutaneously. The pain was soon relieved and the patient rested well during the remainder of the night. During the day the jaundice increased, discoloring his forehead and neck; the quantity of urine was reduced; fæces of a bluish clayey color. Bethesda water was added to the preceding treatment. No anodyne or hypnotic was given at night. Pulse, 70; temperature, 99½°.

Nov. 13th.—In morning learned that patient had not slept and was very restless during the night, was troubled with headache, pulse, 72; temperature, normal; in the evening found pulse, 80; temperature, 101°. Ordered quininæ sulph., gr. xv., one dose, and urethan, gr. xlv, as an hypnotic.

Nov. 14th.—At 3 A. M. was called and found that my patient had not slept. He requested a dose of morphine, and gr. 1-6 was given subcutaneously, which enabled him to rest well during the remainder of the night. At 8 A. M. temperature was 99½°, with no signs of cinchonism. The amount of urine increased, but of a dark brown color. At 8 P. M., pulse, 90; temperature,

102°; color of fæces unchanged. Quinine repeated and morph. sulph., gr. 1/4, with atropia administered as before.

Nov. 15th.—Patient had rested fairly well, said he was feeling better, pulse, 90; temperature, 100 1/2°. Said he had found two hard lumps the size of a pea in his fæces, but these were not seen by writer. In the evening his pulse was 110, and temperature 103°. Advised him to send for his wife, which he refused to do. Appeared nervous, but not incoherent or delirious. Gave quinine and morphine, as before, but was called at 2 A. M., when another doze, gr. 1-6 was given. Urine obtained for examination.

Nov. 16th.—Found my patient up and dressed at 8 A. M, said he felt well, and would go home. Pulse, 90; temperature, normal. Observed that his pupils were dilated and his voice tremulous. At 1 P. M. called on him and found his condition about the same; talked rationally. Pulse, 100, and temperature still normal. The jaundice had greatly increased, extending over shoulders, chest and arms. The liver could be felt below border of ribs, and there was but slight tenderness on pressure. The bile in urine had increased. By treating with acetic acid and evaporating, leucin spheres and tyrosin needles were detected with the microscope.

In the afternoon marked psychical symptoms were developed; hallucinations of sight first appeared, he saw squaws and women in his room, flowers and toy baloons in the air; hallucination of hearing and delusions then developed; delusions were largely of a depressing melancholy character; he feared that people would kill him, that his business and reputation would be ruined. He then held a prolonged conversation with his wife, who was absent. He was very restless and could not be kept in bed, would start suddenly when addressed, but would answer questions rationally. Dr. H. R. Hopkins saw him in consultation with me and recommended hydrarg. sub. chlorid, gr. v., with sodii bicarb., gr. x, every four hours until catharsis was established; also a dram dose of normal liq. ergot, and potasii bromid, gr. xx, every hour to quiet delirium. Several movements of bowels were produced, the fæces contained bile stains and at last became dark, almost black. Although four doses of bromide were given no effect was observed. Pulse at 9 P. M., 120; temperature, 100°.

Nov. 17th.—Patient more incoherent in his delirium, voice thick and tremulous like that of general paretics, exhalted delusions, and staggering gait when he attempts to walk. He becomes more unmanageable, attempts to escape and even attacks attendant. Pulse 130, weak, and temperature below normal, 97½°. Carphologia developed when patient lay in bed. Stimulants, brandy and digitalis, were administered with beef tea and broth, all of which he takes without opposition. Toward evening he would at times fall asleep and stertorous breathing was observed, but he would again awake and become active. His tongue was very dry and brown, urine drawn with catheter was found diminished in quantity, pupils would not respond to light, nausea, vomiting and great thirst. Dr. Burwell was called and observed that patient's strength was rapidly waning, could suggest nothing in the way of treatment except stimulation, and prophesied speedy death. He agreed with me in the diagnosis of acute yellow atrophy. The patient's strength ebbed slowly during the night in spite of all efforts to stimulate the heart, coma-vigil came on, the profound coma, singultus, black vomit due to gastric hæmorrhage, collapse and death at 8 A. M., ten days after the appearance of first symptoms and forty hours after beginning of second stage of disease. During this second stage when atrophy of liver is most rapid, an accurate physical examination was impossible, on account of meteorism, but the liver could not be felt below border of ribs.

The case is interesting on account of its rapid course and the fact that it was ushered in with symptoms of biliary colic. Whether the disease resulted from obstruction of the ductus communis choledochus, or was secondary to some pre-existing disease of the liver cannot be determined. Cases of acute atrophy following prolonged obstruction of bile ducts are on record, but none following acute obstruction have been reported.

